# Microencapsulation of Fluticasone Propionate and Salmeterol Xinafoate in Modified Chitosan Microparticles for Release Optimization

**DOI:** 10.3390/molecules25173888

**Published:** 2020-08-26

**Authors:** Nina Maria Ainali, Eleftheria Xanthopoulou, Georgia Michailidou, Alexandra Zamboulis, Dimitrios N. Bikiaris

**Affiliations:** Laboratory of Polymer Chemistry and Technology, Department of Chemistry, Aristotle University of Thessaloniki, 54124 Thessaloniki, Greece; ainali.nina@gmail.com (N.M.A.); elefthxanthopoulou@gmail.com (E.X.); michailidougeorgia18@gmail.com (G.M.); azampouli@chem.auth.gr (A.Z.)

**Keywords:** chitosan microparticles, modified chitosan, salmeterol xinafoate, fluticasone propionate, chronic obstructive pulmonary disease, sustained release, dissolution enhancement

## Abstract

Chitosan (CS) is a natural polysaccharide, widely studied in the past due to its unique properties such as biocompatibility, biodegradability and non-toxicity. Chemical modification of CS is an effective pathway to prepare new matrices with additional functional groups and improved properties, such as increment of hydrophilicity and swelling rate, for drug delivery purposes. In the present study, four derivatives of CS with trans-aconitic acid (t-Acon), succinic anhydride (Succ), 2-hydroxyethyl acrylate (2-HEA) and acrylic acid (AA) were prepared, and their successful grafting was confirmed by FTIR and ^1^H-NMR spectroscopies. Neat chitosan and its grafted derivatives were fabricated for the encapsulation of fluticasone propionate (FLU) and salmeterol xinafoate (SX) drugs, used for chronic obstructive pulmonary disease (COPD), via the ionotropic gelation technique. Scanning electron microscopy (SEM) micrographs demonstrated that round-shaped microparticles (MPs) were effectively prepared with average sizes ranging between 0.4 and 2.2 μm, as were measured by dynamic light scattering (DLS), while zeta potential verified in all cases their positive charged surface. FTIR spectroscopy showed that some interactions take place between the drugs and the polymeric matrices, while X-ray diffraction (XRD) patterns exhibited that both drugs were encapsulated in MPs’ interior with a lower degree of crystallinity than the neat drugs. In vitro release studies of FLU and SX exposed a great amelioration in the drugs’ dissolution profile from all modified CS’s MPs, in comparison to those of neat drugs. The latter fact is attributed to the reduction in crystallinity of the active substances in the MPs’ interior.

## 1. Introduction

Lung drug delivery is a significant field of research in the handling of chronic inflammatory pulmonary diseases, such as asthma and chronic obstructive pulmonary disease (COPD) [1]. COPD is a life-threatening lung disease characterized by progressive, persistent airflow limitation and airways inflammation leading to excessive production of mucus as well as progressive decline in lung function [2]. It is a multi-system condition comprising, among others, systemic inflammation, oxidative stress, bony muscle malfunction and cardiovascular symptoms [3]. The principal factors causing the appearance of the aforementioned disease include the continual inhalation of toxic particles and fumes among with tobacco smoking, while the polluted atmosphere, age and genetic predisposition also illustrate a catalytic role [4,5].

**Figure 1:**
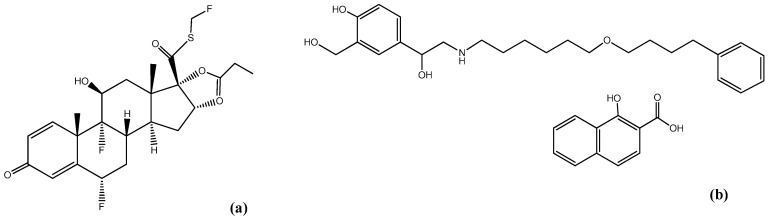
Chemical structures of the used pharmaceutical compounds: (**a**) fluticasone propionate (FLU) and (**b**) salmeterol xinafoate (SX).

The management of COPD requires a multidisciplinary tactic in a risk factor-limiting framework, while patients’ lifestyle demands reconsideration. However, in many cases, the handling of COPD requires the appropriate inhaled medication to enhance the patient’s quality of life through symptom limitation [6]. Regarding pharmacological treatment, inhaled corticosteroids (ICS) is a class of compounds believed to reduce the chronic inflammatory effects in the bronchial area and thus ameliorating limited airflow. Fluticasone propionate (Figure 1a) is a synthetic trifluorinated glucocorticoid which has the role of topical eosinophil inhibitor, administrated for preventing the release of inflammatory agents in the bronchial epithelium [7], as well as for the handling and long-term management of various inflammatory conditions [8,9,10]. However, research studies have occasionally indicated the benefits of co-administrating ICS and long-acting beta2-agonists (LABA), since the mortality in COPD sufferers is diminished, with negligible non-desirable effects [6,11,12,13]. An example of β2-adrenoceptor agonist is salmeterol xinafoate (SX) (Figure 1b), an agent protecting against bronchoconstriction via an airway smooth muscle relaxation pathway [14,15]. In more detail, salmeterol xinafoate is a selective long-acting β2-agonist with a period of bronchodilation lasting more than 12 h [16]. It is a salt existing in two crystalline polymorphic forms, I and II, displaying an enantiotropic correlation [17]. As mentioned above, the combination of β2-agonists such as salmeterol xinafoate with inhaled corticosteroids enhances lung function while controlling the severity of symptoms and the number of acute exacerbations due to the synergistic effect of the two agents [18,19].

The assets of inhalation therapy are abundant and include the decrease in undesirable side effects as a result of the reduced systemic action. In addition, inhalation of drugs by the respiratory route dwindles dosage frequency, while ensuring a sustained release in lung tissue. However, pulmonary delivery efficiency of particles depends on various parameters including the type of formulation, the composition, and the inner shape of the particles along with their aerodynamic diameter [1]. It is proven that particles with size ranging between 1 and 5 μm perform as the finest system to attain successful pulmonary deposition, since particles with a diameter larger than 10 μm deposit in the oropharynx region whereas smaller particles could easily be exhaled [20]. Several classes of polymeric materials, e.g., poly(lactic-co-glycolic acid) (PLGA), poly(ethylene glycol) (PEG) and chitosan (CS), have been suggested as candidate microcarriers for pulmonary drug delivery systems [21]. However, PLGA and PEG have limitations concerning the type of active compounds that can be encapsulated and further released in the target area of the lungs. In contrast, due to its amphiphilic character, chitosan can accommodate both hydrophobic and hydrophilic drugs [22]. Concerning specifically the inhalation delivery of SX and fluticasone propionate (FLU), many research groups studied the amelioration of their co-administration utilizing polymeric excipients. Murnane et al. produced SX and FLU microcrystals by antisolvent crystallization utilizing poly(ethylene glycol), resulting in particles appropriate for inclusion in dry powder inhaler (DPI) formulations [23]. Westmeier et al., examined the formulation and aerodynamic properties of combined particles containing the two APIs via a precipitation pathway, while the obtained suspension was spray-dried afterwards [24]. However, in most cases, the co-administration of both APIs utilizing polymeric excipients does not ensure the release enhancement of these hydrophobic drugs, and therefore, innovative strategies such as improved encapsulation procedures are desired; this concept was studied in the present work.

**Figure 2:**
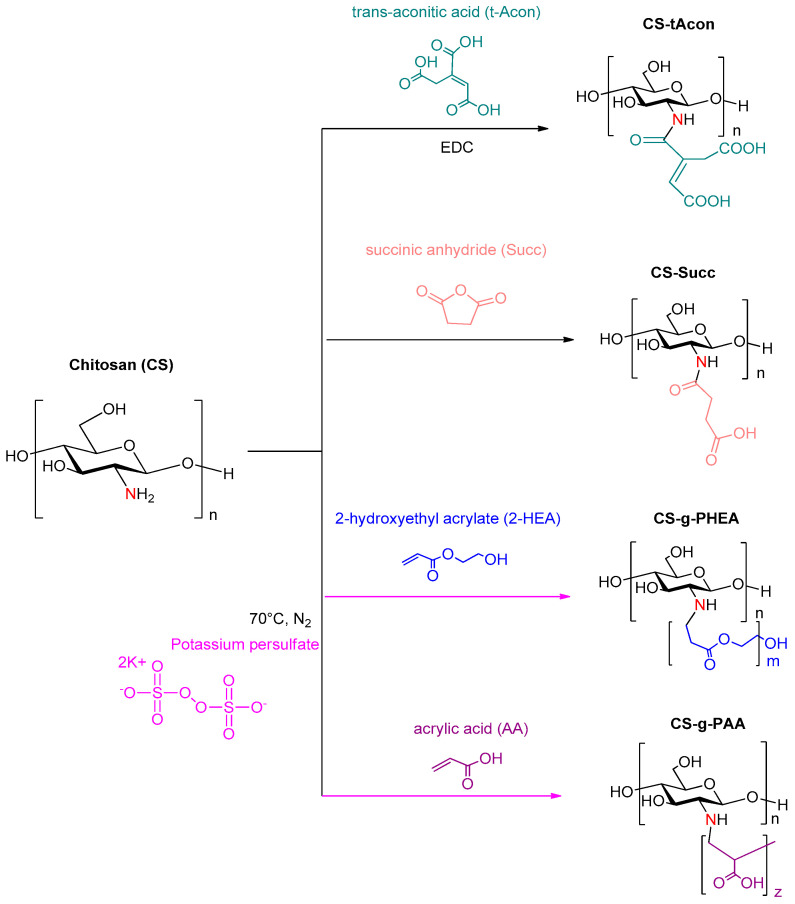
Synthetic routes of chitosan modifications with trans-aconitic acid, succinic anhydride, 2-hydroxyethyl acrylate and acrylic acid.

Generally, chitosan (CS) is a cationic linear polysaccharide [25], produced by chemical or enzymatic deacetylation of chitin. The deacetylated product is a biocompatible, mucoadhesive, biodegradable and non-toxic polymer with constitutional antibacterial properties [26,27]. Concerning its physicochemical properties, chitosan shows efficient solubility in weak aqueous acid solutions, whereas it remains insoluble in neutral and alkaline environments as well as in many organic solvents. This limitation can be improved by chemical modification. The CS macromolecular backbone has a large number of hydroxyl and amino groups, where chemical modification can be carried out. Among the various methods of modification (carboxymethylation, thiolation, succinylation) [28,29], graft copolymerization has been the most applied by researchers, as it allows the formation of functional derivatives by covalent binding of a molecule onto chitosan matrix [30]. In particular, grafting of a polymer’s structure by adding carboxylic groups, i.e., trans-aconitic acid and succinic anhydride [31,32,33,34], or vinylic monomers, i.e., acrylic acid and 2-hydroxyethylacrylate ester [35] (Figure 2) via radical polymerization, enhances its complexing capacity and results in improved swelling ratio, hydrophilicity and bacteriostatic characteristics, without affecting other significant properties. In fact, via radical polymerization, CS is grafted with short oligomeric chains, leading to derivatives with a short number of repetitive units.

Various techniques have been used to formulate chitosan-based microparticles including ionotropic gelation, spray drying or self-assembly [36]. Amongst these methods, ionotropic gelation is generally adopted due to its comparative simplicity, its convenience and mainly owing to the use of non-toxic solvents or excipients and the avoidance of high temperature [37,38]. The simplicity of the technique also renders feasible the encapsulation of hydrophobic compounds during the formulation of the nanoparticles, while many groups have examined the encapsulation of numerous hydrophobic substances in their interior [39]. Furthermore, various polyanions have been reported in the literature for the preparation of chitosan microparticles; however, sodium tripolyphosphate (TPP) is the preferred one. Koukaras et al. [40] demonstrated that the size of the resulting particles relies on the quota between chitosan and the used polyanion. The entrapment of active substances, which are mainly compounds of high crystallinity, in chitosan microparticles results in their amorphization, thus improving their bioavailability.

In the present study, the basic purpose was the design of effective biocompatible carriers for SX and FLU poorly water-soluble drugs for pulmonary delivery, as well as the enhancement of their in vitro release properties and bioavailability. For this purpose, modification of chitosan was performed by grafting hydrophilic groups of different compounds onto CS polymeric backbone in order to ameliorate its inherent properties. Four different materials were synthesized in total, using trans-aconitic acid (t-Acon), succinic anhydride (Succ), 2-hydroxyethyl acrylate (2-HEA) and acrylic acid (AA) as grafting agents (Figure 2). Furthermore, chitosan and modified CS microparticles were formulated via the ionic gelation technique, and SX and FLU were successfully incorporated in the interior of the microparticulate systems, implemented for the first time. Hence, particles with diameter sizes of 1–2 μm were attempted for the respective purposes.

## 2. Results and Discussion

The results will be discussed in two parts. The first part focuses on the synthesis and characterization of chitosan derivatives as well as on the investigation of the evolved interactions between the used monomers and the chitosan backbone. In the second part, the evaluation of these derivatives as appropriate carriers of salmeterol and fluticasone drugs, in the form of particles, is examined. Additionally, emphasis is given to the interactions between the APIs and the polymer matrices, as well as to evaluate the effect of other physical characteristics of derivatives and physical state of the drugs in MPs on drug release procedures.

### 2.1. Characterization of Modified-CS Systems

It is a well-known fact that both carboxylic and hydroxyl groups can ameliorate the hydrophilicity of a material, which is very important for drug delivery systems. One of the principal aims of the present study was the modification of chitosan backbone by introducing –OH and –COOH groups [30,41,42,43]. For this reason, four different monomers—trans-aconitic acid, succinic anhydride, 2-hydroxyethyl acrylate and acrylic acid—were tested as candidates for chitosan modification.

**Figure 3:**
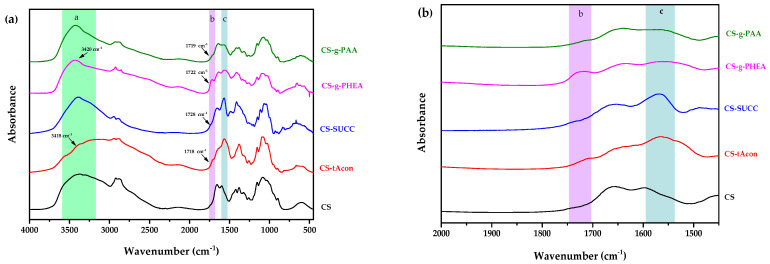
FTIR spectra of the synthesized chitosan derivatives in the range of (**a**) 4000–450 cm^−1^ and (**b**) 2000–1450 cm^−1^.

The structures of the modified chitosan derivatives were studied by FTIR spectroscopy. As can be noticed (Figure 3a), the main bands of neat CS are recorded at 3000–3600 cm^−1^ (a broad band attributed to –OH with maximum at 3457 cm^−1^ and a shoulder at 3200–3270 cm^−1^ due to –NH_2_ stretching, area a), 1659 cm^−1^ (amide I), 1585 cm^−1^ (amide II, area c), 1419 cm^−1^ (C–H and O–H vibrations), 1152 cm^−1^ (anti-symmetric stretching of the C–O–C bridge) and finally at 1078 cm^−1^ (skeletal vibrations involving the C–O stretching), which are characteristic of its polysaccharide nature [44]. Regarding the modified derivatives, most of the typical peaks of neat CS are recorded similarly in their spectra. As depicted in Figure 3b, new peaks are located at 1700–1740 cm^−1^ (b area) owing to the addition of carboxylic and ester derivatives on the chitosan backbone; a fact indicating the effective modification of chitosan, whereas the decrease in intensity of the peak located in 1657 cm^−1^ demonstrated the N-position reaction. Additionally, a small shift was recorded in chitosan’s amino groups from 3255 to 3260 cm^−1^ to about 3240 to 3250 cm^−1^, proving that these groups interacted with the added carboxyl groups. In more detail, concerning the formulated derivatives individually, many comments could be created. t-Acon possesses several peaks, with the most representative being those between 1727 and 1700 cm^−1^ owing to the –COOH fragments. CS-tAcon derivative exhibits the carbonyl group peak at 1718 cm^−1^, implying the successful introduction of t-Acon group to the CS structure [45]. In parallel with the previous data, CS-Succ displays a new peak at 1728 cm^−1^ matching to the free carboxyl groups after the modification of –NH_2_ CS groups with succinic anhydride, while the successful modification of CS chains is revealed by the formation of new amide bonds [46]. For CS-g-PHEA, a relatively strong peak at 1722 cm^−1^ corresponding to ester moieties is visible. Herein, the sharper peak exhibited at 3420 cm^−1^ is attributed to the additional, due to the modification, hydroxyl groups of the derivative, while peak shifting in comparison to the acrylic monomer (Figure S1) is attributed to the hydrogen bonds formed between the amino and hydroxyl groups of CS and the ester groups of HEA units [35]. Finally, as for the CS-g-PAA derivative, the bands observed at 1646 and 1573 cm^−1^ correspond to amide I and II, respectively, while a new shoulder detected at 1719 cm^−1^ reveals the evolved interactions taking place between the amino groups of neat CS with the carbonyl groups of the acrylic acid [47]. For a further confirmation of the successful preparation of the four CS derivatives, the samples were subjected to nuclear magnetic resonance (NMR) spectra.

**Figure 4:**
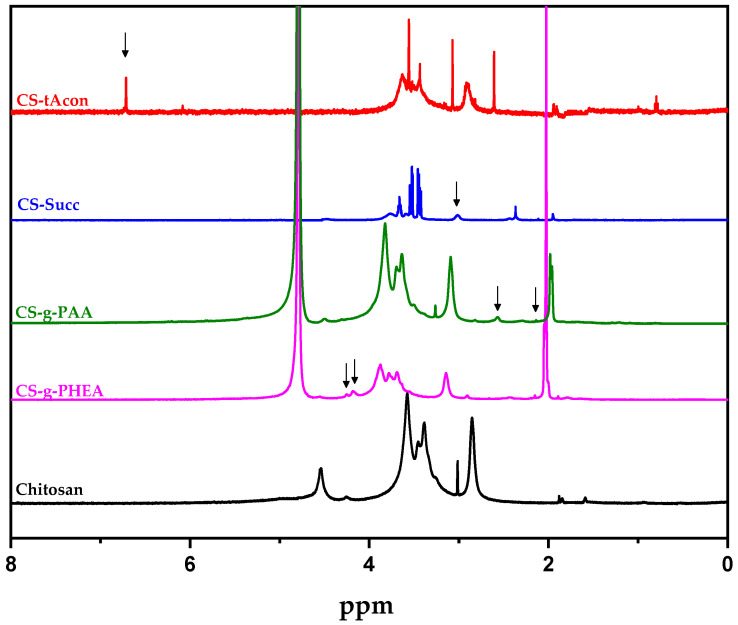
^1^H-NMR spectra of the synthesized chitosan derivatives.

In agreement with FTIR, ^1^H-NMR spectra confirmed the successful grafting of chitosan. Figure 4 illustrates the recorded data of pure CS as well as the four produced CS derivatives. Briefly, in the case of CS-tAcon, the presence of the acid is evidenced by the peak at 6.72 ppm. This peak is attributed to the vinylic proton of trans-aconitic acid. The methylene group of the acid cannot be noticed separately because it overlaps with the chitosan protons at 3.6–3.9 ppm [32]. Concerning CS-Succ, the successful modification is demonstrated by the peak at 3.02 ppm, representing the two slightly differentiated CH_2_ groups from the insertion of the succinic groups. The intensity of this peak indirectly indicates the degree of succinylation of the final material, as Golysev et al. reported [48]. In addition, the effective grafting of chitosan with 2-hydroxyethyl acrylate ester (CS-g-PHEA) is proved. The hydrogen atoms of CS main structure, like methylene protons, present signals around 3.5–3.8 ppm. Consequently, the signals present in the CS-g-PHEA derivative at 4.18 and 4.25 ppm are attributed to CH_2_ protons of the hydroxyethyl group, confirming the successful formulation of CS-g-PHEA, as Mun et al. reported in a recent study [49]. Finally, the formation of chitosan grafted-copolymer with acrylic acid (CS-g-PAA) is evidenced by the presence of peaks at 2.1 and 2.6 ppm, corresponding to the CH_2_ and CH protons of the acrylic acid backbone, respectively [35]. The peak at 4.8 ppm present in all the ^1^H-NMR spectra is related to the used solvent.

The grafting percentages (GP) as well as the grafting efficacy (GE) of all the materials were calculated according to Equations (1) and (2). The GP specifies the increase in CS weight when subjected to grafting while GE indicates the ability of the monomer to graft onto the CS backbone, as calculated from the weight of the grafted monomer. The synthesis yields (%) were relatively high for all materials. More specifically, concerning the CS-tAcon derivative produced with the aid of EDC, providing the activation of the carboxylic groups of trans-aconitic acid, the GP was 21% while its GE was 84%. N-succinyl-grafted-CS (CS-Succ) derivative was prepared by a ring opening reaction of chitosan with succinic anhydride, a cyclic anhydride. The CS-Succ modification’s GP was 2.5%, whereas its GE was 16.7%. Concerning the acrylic derivatives, the preparation of CS derivatives with short oligomeric chains grafted on CS backbone was intended. Consequently, short-time free radical reactions took place, avoiding the extended PHEA and PAA chains. Regarding CS-g-PHEA derivative, its GP was calculated at 19%, thus the GE was 76%. Finally, concerning CS-g-PAA material, the resulting GP was 8%, while its GE was 73.4%. Regarding the acrylic derivatives, a similar grafting behavior is observed, whereas CS-tAcon and CS-Succ demonstrate a rather different intermediate comportment. CS-tAcon revealed a superior grafting percentage compared to CS-Succ, probably attributed to the presence of three carboxylic groups on the monomer’s structure in comparison to the succinic anhydride. 

**Figure 5:**
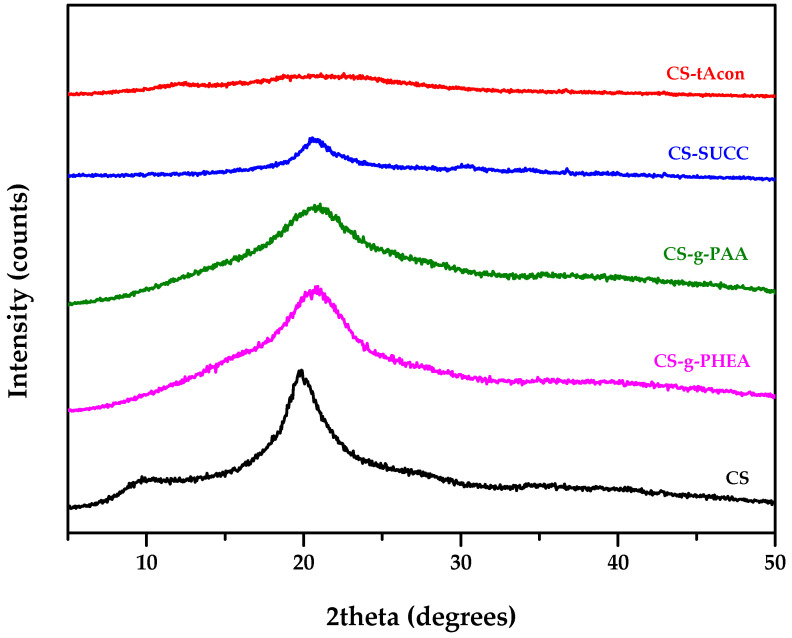
X–ray diffraction (XRD) patterns of CS, CS-g-PHEA, CS-g-PAA, CS-Succ and CS-tAcon derivatives.

The crystalline phase of the prepared CS-modified derivatives was investigated using XRD (Figure 5). Regarding neat CS, the XRD pattern exhibited an amorphous region with two quite broad peaks at 2θ = 11° and 20°, which are the typical fingerprints of the semi-crystalline character of the studied polymer [35,43,44]. Regarding the prepared grafted CS derivatives, XRD data displayed that all derivatives are almost amorphous, since only one broad peak was recorded at approximately 2θ = 21°. The abovementioned peak is wider and slightly shifted in comparison with CS’s diffractogram, revealing that the inclusion of the added groups into the CS backbone affected its semi-crystalline character. This was expected since the addition of small molecules on the polymeric chains of CS decreases its folding ability, and thus, the crystal structure formation. Analogous results were found in our previous works on CS derivatives [31,32,35,44,46].

**Figure 6:**
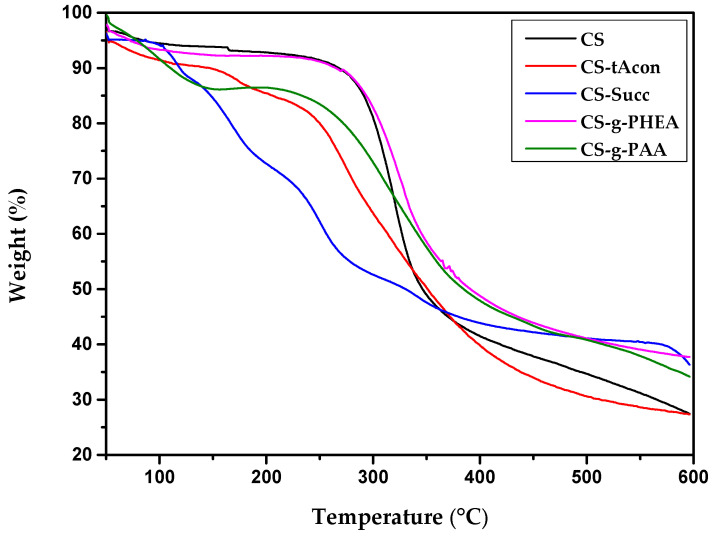
Thermogravimetric analysis of prepared: CS-tAcon, CS-Succ, CS-g-PAA and CS-g-PHEA derivatives.

Following the above XRD analysis, TGA curves were recorded in order to examine the effect of the modifications on the thermal stability of chitosan. The thermal degradation of chitosan could be described as a two-stage procedure. Briefly, in the case of neat chitosan, the first step occurred at temperatures between 50 and 180 °C corresponding to a mass loss of 8–10 wt% associated with the loss of absorbed or bound water. Indeed, at this temperature range, a collapse of the strong hydrogen bonding between active groups (amine, hydroxyl) of chitosan backbone and water molecules occurs [50]. The second step was reported in the temperature range of 180–410 °C and was linked to the chitosan degradation and, to a small extent, to its deacetylation [51]. Concerning the derivatives’ thermograms (Figure 6), it is obvious that the change in the structure of the polymeric matrix, by the addition of new active carboxylic groups, affects the thermal character of the produced material, since the thermal profile of the formulated modified structures indicates a multiple-step mass loss. In particular, the degradation profile of CS-tAcon and CS-Succ showed different curve patterns with smaller, multiple mass loss stages, showing a gradual decomposition. Moreover, CS-g-PAA exhibits another profile. The first stage starts at 50 °C and continues up to 130–140 °C, during which there was 7–10% mass loss, associated to the unbound water. The second stage from 190 to 300 °C and the third stage from 310 to 600 °C may be related to the degradation of chitosan and the decomposition of different structure of the grafting product, respectively [52]. The case of CS-g-PHEA seems interesting, as the derivative displays a degradation profile similar to that of chitosan. In all cases, the derivative materials demonstrate reduced thermal stability in comparison to neat chitosan, while the thermal degradation of the polymer backbone outsets at a lower temperature range. This observation is attributed to the susceptibility to abstraction of carboxyl and carboxyethyl groups in comparison to amino groups of CS, as was reported in an analogous study of Metzler et al. [53]. Finally, the percentage of mass residue at 600 °C is high, typical for CS materials, while the mass residue is observed at a greater percentage in CS derivatives’ thermograms, attributed to the addition of the supplementary groups.

**Figure 7:**
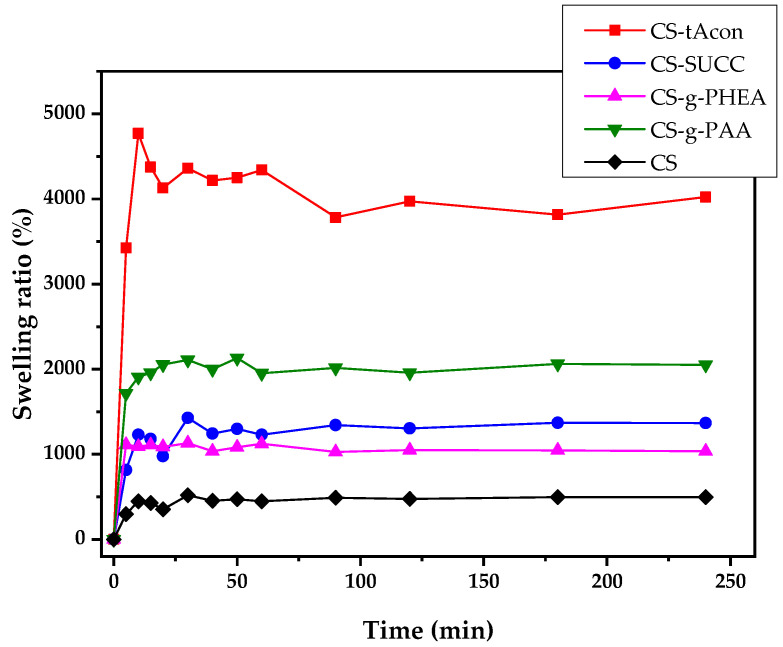
Degree of swelling vs. time of CS, CS-tAcon, CS-Succ, CS-g-PHEA and CS-g-PAA derivatives.

One significant characteristic of polymeric systems destinated for pharmaceutical purposes is their inherent property of swelling. Polymeric networks can be swollen due to alterations in their external environment. Additionally, polymeric materials containing hydrophilic groups swell to a higher degree compared to those containing hydrophobic groups since the chemical structure of a polymer directly affects its swelling capacity [45]. As a result, hydrophobic groups lead to a breakdown in the appearance of water [54]. In fact, the swelling of a polymer is basically forced by electrostatic repulsions between functional groups with similar charges. Figure 7 presents the degree of swelling data for neat chitosan, CS-tAcon, CS-Succ, CS-g-PHEA and CS-g-PAA in their sponge form after freeze-drying, in regard to the swelling time, at pH = 7.4. It can be observed that efficient hydrophilic materials have been prepared with high gelling ability, since in all materials the swelling degree ranged from 1000 to 4000%. Matrix swelling of the prepared materials is much higher than that of neat CS. Regarding neat CS, it exhibits a low degree of swelling ranging between 50 and 200%, mainly affected by the degree of deacetylation, molecular weight and pH [55]. In a further step, as shown in Figure 7, the four studied derivatives of CS reveal a two-phase swelling profile, comprised of an initial burst swelling phase (fast water uptake) followed by an erosion stage leading lastly to a steady swelling phase in all materials. After comparison of the four different derivatives, CS-tAcon showed, as expected, the highest degree of swelling, since the degree of grafting, and thus the number of the added characteristic –COOH groups, is greater in comparison to the other materials. 

### 2.2. Characterization of MPs

Microparticles were formulated via ionotropic gelation technique, with sodium tripolyphosphate utilized as physical cross-linker. In detail, the MPs were prepared as a result of the ionic interactions between the negative charged TPP groups and the cationic amino groups of CS. Relative projects studying the aforementioned technique have been reported previously by our research team, including the synthesis of nanoencapsulated budesonide particles for pulmonary delivery [56], deferoxamine mesylate-laden CS nanoparticles for iron chelator release [57], timolol maleate-loaded particles for ocular delivery [31] as well as paliperidone incorporation in polymeric carriers for intranasal delivery [58].

A significant factor affecting the final size of the formulated particles is the ratio between chitosan and TPP. Previous studies determined the correlation among the CS/TPP ratio and the size of the prepared particles, proper for each different application [31]. The control of microparticles’ size is crucial, as it affects the degradation rate of the polymeric matrix and the entrapment percentage as well as the release profile of the active substances. Moreover, since SX and FLU are mainly used for inhaled delivery systems, the CS/TPP ratio was optimized in order to obtain particles ideal for lung delivery. As has been reported, microparticles greater than 5 μm are mainly accumulated in the oropharynx region [59], smaller particles <0.5 μm are easily exhaled, whereas microparticles 1–5 μm in size are amassed in the upper respiratory system. In the framework of the present study, the formulated particles should fluctuate between 1 and 5 μm to achieve efficient pulmonary deposition [20]. 

Herein, blank particles with different CS/TPP ratios were synthesized and characterized via dynamic light scattering (DLS) analysis in order to select the appropriate ratio for the specific application. The studied CS/TPP *w*/*w* ratios were 2/1–7/1. As shown in Table 1, in all blank particles, the appropriate ratio between the used polymer and the crosslinker, leading to larger sizes, differentiates. In the case of neat CS, the larger particles were obtained for the 4/1 ratio, formulating particles with 450 nm size. This outcome is in contrast with Koukaras et al.’s results [39]. This difference is attributed to the different molecular weight of CS as well as to the different experimental conditions, namely faster stirring speed during the preparation. Regarding the CS derivatives, the larger particles were attained for CS-tAcon/TPP 7/1, CS-Succ/TPP 5/1, CS-g-PHEA/TPP 6/1 and CS-g-PAA/TPP 6/1 ratios. It could be observed that in the case of acrylic modifications, the bigger particles are obtained with the same polymer/crosslinker ratio, whereas for CS-tAcon the particles size is almost proportionately affected by the abovementioned ratio. Finally, for CS-Succ modification, a similar behavior to the CS-tAcon derivative is observed. 

Among the different CS/TPP ratios, the 6/1 ratio resulted in the largest particles in most cases (Table 1); it was therefore chosen for the formulation of drug-loaded particles. The prepared blank particles are in the nanometric scale and thus are too small for delivery by inhalation; however, upon loading with FLU and SX, the obtained drug-loaded particles are bigger and thus more appropriate for inhalation administration. This observation is in agreement with the literature [60,61]. More specifically, as presented in Table 2, the DLS results indicate that the size of drug-loaded microparticles ranges approximately between 1.2 and 2.2 μm for all CS derivatives except CS-g-PAA-TPP. The diminished size is probably attributed to the presence of the grafted polyacrylic acid moieties. In the literature, nanoparticles formulated with CS and polyacrylic acid have been studied extensively. It was observed that at pH 4.5, the carboxylic and amino groups are both partially ionized and consequently, able to interact between each other, formulating polyelectrolyte complexes [62]. This effect, in addition to the presence of TPP, results in CS-g-PAA particles with nano-scale diameter. Furthermore, the results given in Table 2 reveal a correlation between the percentage of the encapsulated drug and the size of the arising microparticles. More specifically, increasing the amount of the added drug results in larger particle size. The increment of concentration results in a higher percentage of drug encapsulation and eventually, the formation of bigger microparticles. Moreover, the presence of the active compounds burdens the interaction between the polymeric matrix and the polyanion groups, additionally leading to greater size of the formulations. This observation is in agreement with the outcome from a previous work from our team where budesonide was encapsulated in CS nanoparticles and the size of the formulated particles increased while increasing budesonide’s concentration [56]. Modification of the CS backbone, in most cases, significantly affects the size of the resulting particles. The addition of new compounds in the polymer’s structure results in the augmentation of hydrophobic interactions, affecting the final size of the formulated particles. 

Zeta potential is a factor indicating the surface charge of the nanoparticles in the colloidal system impacting its stability [63]. Microparticles with zeta potential values lower than −30 mV or higher than +30 mV are characterized as stable colloidal systems and do not form aggregates [64]. SX and FLU are two active compounds with neutral charge [65,66]. Consequently, the positive surface charge of the prepared microparticles is attributed solely to the polymeric matrixes. Zeta potential is affected by the presence of the added carboxylic and hydroxyl groups in the CS modified backbone, leading to lower zeta potential values due to the presence of anionic charges. Despite the added anionic groups, the positive surface charge of the formulated microparticles is attributed to the remaining free unreacted amino groups, since after the preparation of the CS derivatives, free amino groups are present in the polymeric backbones. Moreover, the reduction in the positive zeta potential values is an additional indication of the successful grafting of the monomers or the acrylic oligomers on free amino groups. Interestingly, CS-tAcon microparticles reveal the lowest zeta potential values among the samples. This is attributed to the enhanced grafting percentage as well as to the presence of three carboxylic groups in trans-aconitic acid monomer, leading to a CS derivative with increased anionic charge. Nevertheless, in all cases, the zeta potential value reveals stable colloidal systems of the resulting microparticles. 

**Figure 8:**
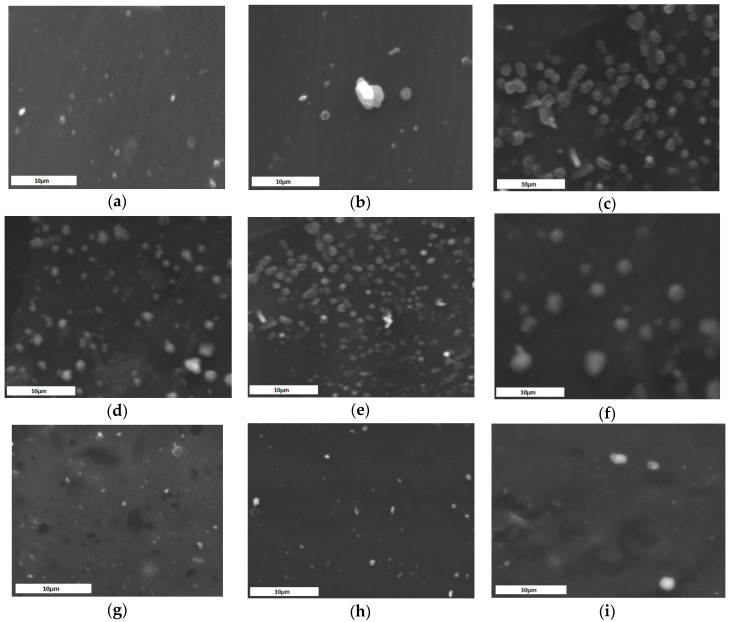
SEM image of: (**a**) CS-tAcon-sodium tripolyphosphate (TPP)-10% FLU/SX, (**b**) CS-tAcon-TPP-20% FLU/SX, (**c**) CS-tAcon-TPP-30% FLU/SX, (**d**) CS-Succ-TPP-10% FLU/SX, (**e**) CS-Succ-TPP-20% FLU/SX, (**f**) CS-Succ-TPP-30% FLU/SX, (**g**) CS-g-PAA-TPP-10% FLU/SX, (**h**) CS-g-PAA-TPP-20% FLU/SX and (**i**) CS-g-PAA-TPP-30% FLU/SX.

SEM was further utilized to examine the morphological characteristics of the obtained microparticles. As can be seen in Figure 8a–i as well as in Figure S2a–f, the size of the formulations is in agreement with DLS measurements. As depicted in SEM micrographs, in any ratio between the active substances and the polymer matrix, the final samples are formulated as individual particles of spherical shape with smooth morphology in most cases. 

**Figure 9:**
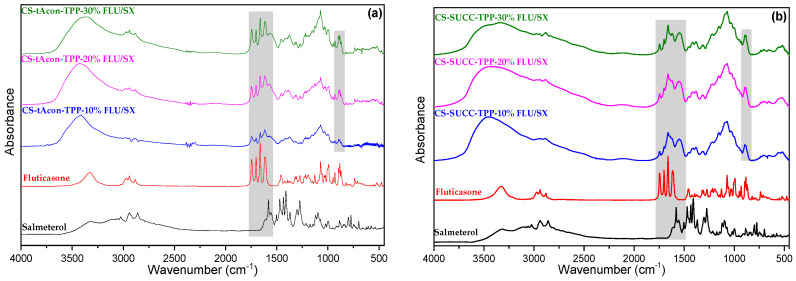

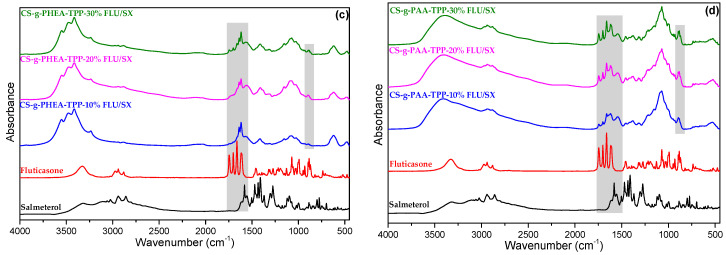
FTIR spectra of (**a**) CS-tAcon, (**b**) CS-Succ, (**c**) CS-g-PHEA and (**d**) CS-g-PAA salmeterol xinafoate and fluticasone propionate-loaded particles containing drug in different ratios 10%, 20%, 30% *w*/*w*.

FTIR analysis was utilized again to further study the ionic interactions between CS, CS derivatives and TPP, while the dynamic hydrogen bond formation among the polymeric network and drugs in the microparticles’ interior (Figure S3 and Figure 9a–d) was also examined. Regarding CS and CS derivatives’ spectra, all the characteristic peaks of them were observed. Nevertheless, consistent with previous works, particles formulated with TPP polyanion via an ionic gelation technique show shifts in their FTIR spectra in the region of amides (amide I and II) and phosphate groups of TPP, with the latter recorded at 897 cm^−1^ [67]. Not only for CS/TPP laden particles, but also for CS derivatives’ formulations, the abovementioned peak was accordingly shifted to lower wavenumbers indicating the interactions between amino and phosphate groups. From APIs’ perspective, neat SX displays its principle peaks at 1409 cm^−1^ (bend for hydroxylic group), 1580 cm^−1^ (>NH), 1650 cm^−1^ (C=C, aromatic ring), 883 cm^−1^ (C–O for ether group), 1080 cm^−1^ (C–O–C), 884 cm^−1^ (C–H bend for aromatic benzene ring), and 1320 cm^−1^ (C–N stretch for aromatic ring), whereas fingerprints of FLU are observed at 1661 cm^−1^ (C=C), 1409 cm^−1^ (–OH), 991 cm^−1^ (S–H), 1024 cm^−1^ (C–F), 883 cm^−1^ (C–O for ether group), 1715 cm^−1^ (C=O for ketones) and 3000 cm^−1^ (C-H for aldehyde). In SX and FLU-encapsulated microparticles, the detection of any alteration to –OH absorbance band is not achievable since the peak presenting the CS’s hydroxyl groups is wide and may overlap the corresponding peak of the drug formulations. Nevertheless, there is a small shift of the peak absorption of C=O of FLU from 1743 to 1738 cm^−1^, while the other peaks at 1650 cm^−1^ for alkenes C=C remains stable. With a careful consideration, in the range between 1658 and 1740 cm^−1^, small peaks are observed in all microparticles as shoulders; peaks absent in neat CS and CS derivatives. The aforementioned shifts and new peak absorbances may emerge because of the interactions of API’s groups with –NH_2_ or –OH of CS, probably via hydrogen bonding.

**Figure 10:**
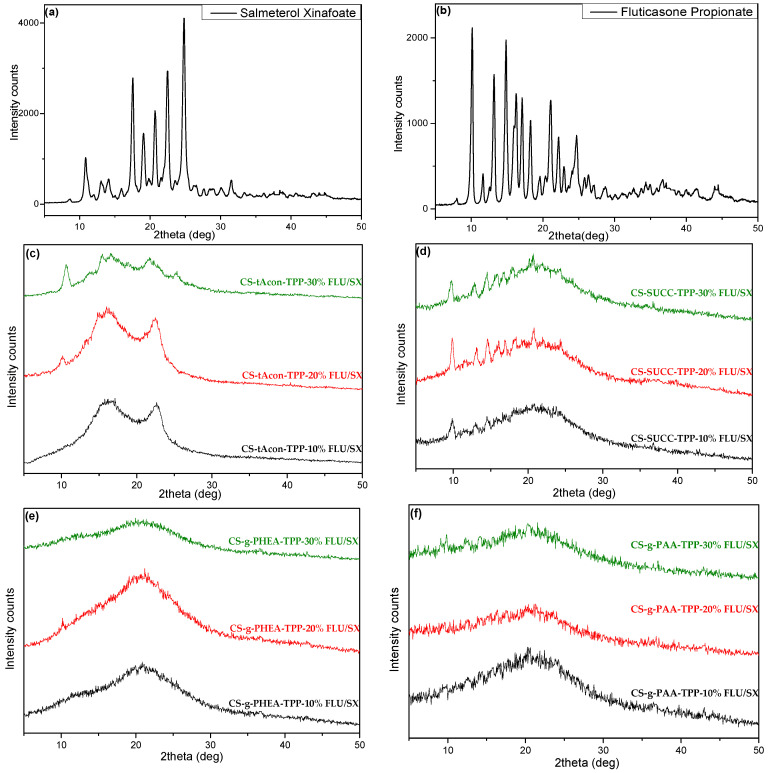
XRD diffractograms of neat (**a**) salmeterol xinafoate, (**b**) fluticasone propionate and XRD diffractograms of particles loaded with salmeterol xinafoate and fluticasone propionate in different ratios, 10%, 20%, 30% *w*/*w*, of (**c**) CS-tAcon, (**d**) CS-Succ, (**e**) CS-g-PHEA and (**f**) CS-g-PAA.

Figure 10c–f presents the XRD diffractograms of the prepared microparticles and the used drugs in order to examine the physical state and the interactions of salmeterol and fluticasone in microparticles’ interior. As referred above, CS is a semi-crystalline polymeric material with two typical peaks at 2θ = 11° and 21°. On the other hand, the two APIs are highly crystalline compounds, as clear XRD peaks appear at 2θ = 10.9°, 13.1°, 14.1°, 17.5°, 19.1°, 20.6°, 22.5°, 24.8° and 10.2°, 11.6°, 13.1°, 14.8°, 16.1°, 17.0°, 18.2°, 19.7°, 21.0°, 22.1°, 23.1° and 24.7° for SX and FLU, respectively (Figure 10a,b) [17,24]. XRD patterns of the formulated CS microparticles showed that both salmeterol and fluticasone are probably incorporated in their crystalline form (Figure S4). In brief, in all prepared formulations, there are some sharper peaks present at 10.2°, 13.2°, 14.9°, 16.4°, 17.2°, 18.3°, 20.9°, 22.5°, and 24.8°, while the relative intensity of CS peaks is diminished. Comparing these peaks with neat drug patterns, it is very difficult to distinguish if both drugs are in crystalline form or if one of them is in amorphous phase, since both drugs have peaks at almost the same positions (Figure 10a,b). However, their intensities are completely different. FLU has a high intensity peak at 10.2°, while in SX the sharpest peak was recorded at 24.8°. Thus, based on these, it is possible that FLU was encapsulated in its crystalline form in CS microparticles, while SX was amorphous. Furthermore, a reduction in the crystalline phase of CS is also interpreted by the reposition of inter and intramolecular polymeric network owing to the crosslinking procedure with the TPP ions. Additionally, the proportionate reduction in the drugs’ fingerprint peaks after their encapsulation in the polymeric network is noticeable, while the intensity of these peaks lessens as the drug content is reduced. Regarding the microparticles of the modified CS derivatives, a similar behavior can be observed in CS-tAcon-TPP-FLU/SX and CS-Succ-TPP-FLU/SX concerning the peak intensities and their positions as in the case of neat CS. There are some small peaks, mainly at 20 and 30 wt% of encapsulated drugs, which are recorded at the same positions mentioned above for neat CS, and thus it could be concluded that only FLU drug was encapsulated in crystalline form, while SX was encapsulated in amorphous form (Figure 10c,d). In the other derivatives, CS-g-PHEA-TPP-FLU/SX and CS-g-PAA-TPP-FLU/SX, it is clear that such peaks have not been recorded and therefore the drugs have been encapsulated in an amorphous form in both derivatives (Figure 10e,f).

**Figure 11:**
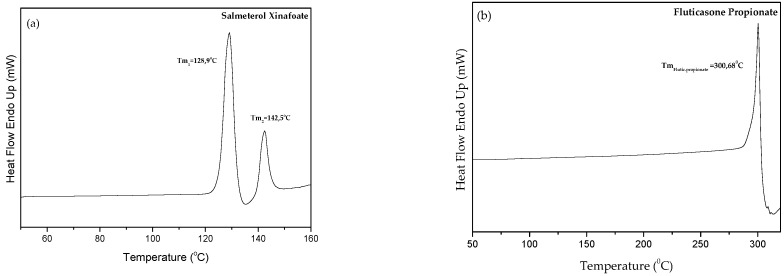
DSC curves of (**a**) salmeterol xinafoate and (**b**) fluticasone propionate.

DSC measurements were additionally performed in order to estimate the physical state of the active substances inside the polymeric microcarriers, namely the developed interactions between them. At first, the examination of the thermal behavior of the drugs was performed. The results given in Figure 11a,b confirm that both salmeterol xinafoate and fluticasone propionate are crystalline compounds. More specifically, SX is a compound which, as reported in the literature, has two characteristic melting endothermic peaks at 128.9 and 142.5 °C, representing the two different types of crystal which it is composed of [65]. Concerning FLU, an endothermic melting peak is observed at 300.68 °C.

**Figure 12:**
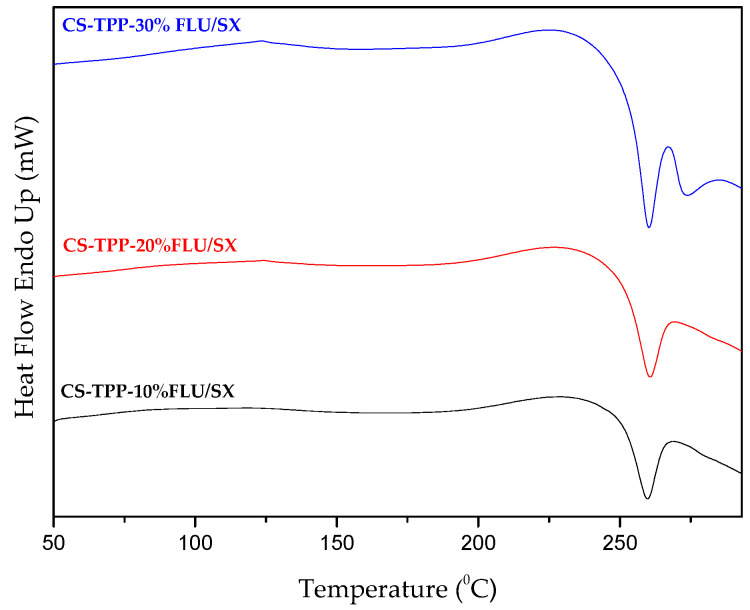
DSC curves of CS microparticles containing FLU/SX in 10, 20 and 30 wt%.

**Figure 13:**
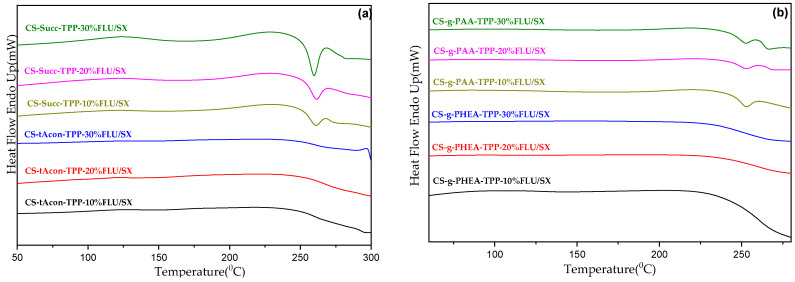
DSC thermograms of (**a**) CS-tAcon- FLU/SX, CS-Succ- FLU/SX and (**b**) CS-g-PHEA- FLU/SX, CS-g-PAA- FLU/SX microparticles.

The crystallinity of the two drugs inside the microparticles interior was further studied by DSC (Figure 12; Figure 13) to complement the XRD results. It is well-known that neat CS does not demonstrate a melting peak visible in DSC analysis [68]. As it can be seen, DSC thermograms are similar for all drug-loaded microparticles. Briefly, CS or CS derivative samples loaded with drugs in all percentages do not reveal any endothermic T_m_ peak concerning the SX drug, confirming that the active substance is in an amorphous state inside the microparticles, a result that agrees with XRD data. Neat FLU presents an endothermic melting peak at 300.68 °C, whereas in all DSC thermographs, an exothermic peak was recorded at 268 °C, which should be attributed to the thermal decomposition of CS. According to TGA data, the decomposition of CS is initiated approximately at 250 °C. Consequently, a melting peak is not detectable for FLU as it is located after the thermal degradation of the polymeric chains of chitosan or its derivatives. 

The encapsulation efficiency (EE %) of SX and FLU in the interior of chitosan and modified chitosan particles is a distinctive parameter which directly influences the release profile inducing an augmented bioavailability [69]. Table 3 outlines the yield (%), drug loading efficacy (%) and encapsulation efficiency (%) of all the prepared particles. Regarding yields, they varied from 29.1% to 50.2%, indicating high process efficacy when the drugs’ content increases in all cases. The aforementioned relation is similar to drug loading efficacy in the majority of samples. In general, the hydrophobic character of both SX and FLU greatly affects their loading efficacy. Hydrophobic interactions take place between drugs’ molecules, contributing to a lower encapsulation efficacy for formulations containing 30 wt% FLU/SX, whereas 10 wt% samples exhibited the higher encapsulation percent.

**Figure 14:**
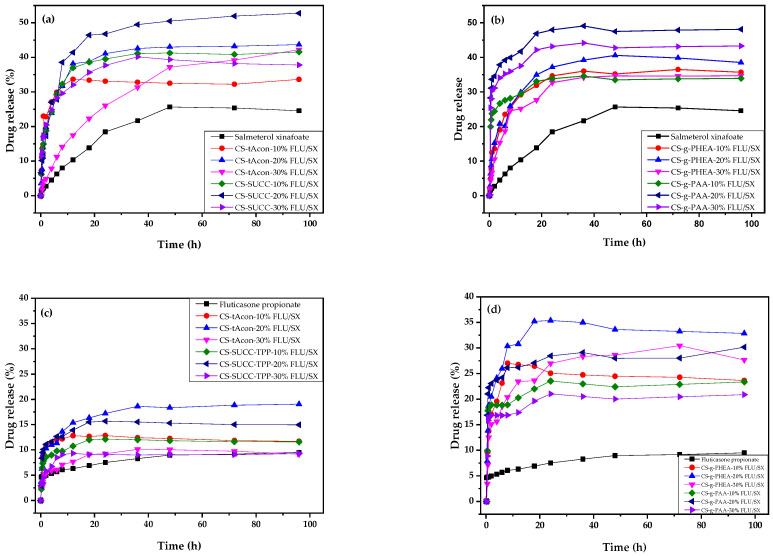
In vitro release rate of salmeterol xinafoate from (**a**) CS-tAcon-FLU/SX, CS-Succ-FLU/SX and (**b**) CS-g-PHEA-FLU/SX, CS-g-PAA-FLU/SX microparticles, and of fluticasone propionate from (**c**) CS-tAcon-FLU/SX, CS-Succ-FLU/SX and (**d**) CS-g-PHEA-FLU/SX, CS-g-PAA-FLU/SX microparticles.

Dissolution studies, in Figure 14, showed that in all cases there is an enhancement of drug release. As can be observed, both drugs exhibited low dissolution rate and extent, reaching only 25 and 9.5% release, for SX and FLU, respectively, even after 100 h. These low release data could be attributed to the inadequate water solubility and high hydrophobic nature of both APIs and are in good agreement with those reported in literature [67,70]. The dissolution of the APIs from CS microparticles (Figure S5) does not ameliorate the release behavior of FLU or SX. Contrary to this, all the modified microparticles’ formulations presented much higher dissolution release rate as well as extent. As it can be seen from Figure 14, both SX and FLU are released from the formulated microparticles following a two-step release profile, comprised of an initial burst release appearing approximately in the first 20–30 h, owing to the surface-bonded active compounds, succeeded by a sustained release profile until the end of the study. This biphasic release profile may be associated with the adsorption on the surface of the active substances and their amorphization (first phase), as well as polymeric structure’s swelling and erosion abilities (second phase). Additionally, the microparticles containing 20 wt% SX and FLU exhibited superior performances regarding drug release. These samples exhibited the higher release rate, reaching almost 50% of SX release (a kind of doubling effect) and 36% of FLU release (almost four times higher than neat FLU). Microparticles containing 30 wt% of drugs presented a poorer release in comparison to 20 wt% samples, due to the possible hydrophobic interactions taking place between the drugs’ molecules or due to the higher amount of drug in the microparticles. Indeed, according to the literature, when hydrophobic active substances are entrapped in polymeric matrices, an inversely proportional relationship between drug concentration and drug release was reported [71,72,73]. When the drug amount becomes higher, the drug release is reduced. 

Regarding the correlation between the modified structures and the drugs’ release of SX and FLU, it is clear that it depends on the encapsulated drug as well as the used CS derivative. For SX, it seems that the drug amount plays the most important role in drug release, since the obtained release percentages are almost similar for all derivatives with very small differences for the same encapsulated amount. This is because, as verified by XRD and DSC, SX was encapsulated in an amorphous form in all microparticles. It is well known that amorphous drugs have up to 1000 times higher dissolution rates than crystalline forms [71,72,73]. In the case of FLU, there are some important differences in the release profiles between the derivatives. As it can be observed in Figure 14c,d, the drug release is much lower in both CS-tAcon and CS-Succ derivatives compared with CS-g-PHEA and CS-g-PAA. This behavior could be attributed to the microencapsulation of FLU in CS-tAcon and CS-Succ derivatives in crystalline form, while it was microencapsulated in amorphous form in CS-g-PHEA and CS-g-PAA. This was confirmed by XRD patterns (Figure 10). It seems that the interactions between the polymeric matrix and FLU are much higher in these derivatives due to the PHEA and PAA macromolecules that formed as grafted chains in CS backbone, which could not only increase drug–network interactions but also the matrix flexibility [74].

## 3. Materials and Methods

### 3.1. Materials

Chitosan was supplied by Kraeber and Co GmbH (Ellerbek, Germany), possessing a molecular weight of 18,000 g/mol and a degree of deacetylation > 94%, as was determined by viscometry and ^1^H NMR, respectively, in our previous study [56]. Trans-aconitic acid, succinic anhydride and 1-ethyl-3-(3-dimethylaminopropyl)carbodiimide hydrochloride (EDC) used in synthesis of carboxylated derivatives were supplied by Aldrich Chemicals Co. (Stainheim, Germany). 2-hydroxyethyl acrylate was purchased from Alfa-Aesar (Karlsruhe, Germany) and the hydroquinone inhibitor was removed by passing 2-hydroxyethyl acrylate, at least twice, through disposable inhibitor–remover packed column supplied from Aldrich, before any use. Acrylic acid and potassium persulfate were purchased from Sigma-Aldrich Co. (Saint Louis, MO, USA). Sodium tripolyphosphate (TPP) used as ionic crosslinker (85% purity) and acetonitrile HLPC purity (ACN) were supplied from Aldrich chemicals (Steinheim, Germany). Fluticasone propionate and salmeterol xinafoate drugs (99.99% purity) were kindly donated by Medicair Bioscience S.A. (Athens, Greece). All other reagents used were of analytical grade.

### 3.2. Synthesis of CS Derivatives

#### 3.2.1. Chitosan Modified with Trans-Aconitic Acid (CS-tAcon)

The synthesis of modified chitosan with trans-aconitic acid (CS-tAcon) was carried out according to Michailidou et al. [44]. Briefly, 1 g of trans-aconitic acid was dissolved in methanol in the presence of EDC (1.15 g EDC per 75 mL MeOH) and then gradually added into a chitosan solution (4 g chitosan in 260 mL acetic acid 2% *v*/*v*). The amount of trans-aconitic acid used was addressed to the 0.5/1 molar ratio of reactive groups COOH/NH_2_. The reaction took place for 12 h at room temperature, under continuous mechanical stirring. The product was frozen, freeze-dried (Scanvac, Coolsafe 110-4 Pro, Labogen Scandinavia) and treated further with Soxhlet extraction using acetone as a solvent for purification and the removal of unreacted reagents. 

#### 3.2.2. Chitosan Modified with Succinic Anhydride (CS-Succ)

The synthesis of CS-Succ was set up as reported by Skorik et al. [75]. In brief, 2 g of chitosan were dissolved in 100 mL of aqueous acetic acid solution (1% *v*/*v*), followed by the addition of 0.3 g of succinic anhydride, diluted in a small volume of acetone. The molar ratio between carboxyl to amino groups corresponded to 0.5/1. The reaction was carried out for 7 h at room temperature. The product was precipitated from the viscous reacting mixture by adding 100 mL acetone and was isolated by filtration. The product was washed thoroughly in 100 mL of an aqueous 3% *w/v* NaHCO_3_ solution. The resulting solution was dialyzed against distilled water for 3 days, freeze-dried and finally a cotton-white material was obtained.

#### 3.2.3. Chitosan Grafted with 2-Hydroxyethyl Acrylate (CS-g-PHEA)

The graft copolymerization of 2-hydroxyethyl acrylate on chitosan was carried out in a two-necked round-bottom glass reactor [48,76]. In a typical grafting method, 10 g of CS were dissolved in 400 mL acetic acid (2% *v*/*v*), and potassium persulfate (KPS) (37.5 mg) was transferred into the reactor and stirred magnetically at 300 rpm for 10 min. Then, 2.5 g of 2-HEA were added dropwise, resulting in a final mole ratio CS/2-HEA 5/1. The grafting reaction was carried out at 60 °C for 4 h under a nitrogen atmosphere. Then, the chitosan derivative was washed several times with deionized water, and freeze-dried under reduced pressure at −60 °C so as to obtain the cotton-like final product (CS-g-PHEA). The product was purified using a Soxhlet apparatus and methanol to remove the unreacted HEA and PHEA homopolymer formed during the reaction.

#### 3.2.4. Chitosan Grafted with Acrylic Acid (CS-g-PAA)

For the preparation of chitosan grafted with acrylic acid derivative [46], 10 g of CS were initially dissolved in 2% *v/v* aqueous acetic acid solution, followed by the addition of AA solution. The amount of acrylic acid used (1 mL) corresponded to 1:2 molar ratio of reactive groups COOH/NH_2_. Then, KPS (0.16 g) was added as an initiator. The final solution was poured into a 1000 mL flask, and then placed in a thermostated water bath at 60 °C for 2 h under a nitrogen atmosphere. After rapid cooling down to room temperature, a purification stage ensued by extraction with methanol in a Soxhlet apparatus for 3 days, allowing the removal of the unreacted acrylic acid, initiator and the formed homopolymer. 

### 3.3. Preparation of MPs via Ionic Gelation

Chitosan and modified CS particles were prepared according to the widely spread ionotropic gelation technique, utilized frequently and extensively by our group [31,56,57]. Briefly, blank particles were obtained upon the dropwise addition of TPP aqueous solution to a CS acetic acid 2% *v/v* solution, under magnetic stirring. According to Koukaras et al., the CS/TPP ratio is critical and tailors the size as well as the size distribution of the prepared particles [39]. Thus, different concentrations of CS and TPP were applied to control the CS/TPP ratios, providing stable particles with sufficient size, appropriate for the target of this study (CS/TPP ratios 2/1, 3/1, 4/1, 5/1, 6/1 and 7/1 *w*/*w*). The particles were kept in stirring for 4 h and centrifuged at 11,000 rpm for 20 min (Heraeus™ Pico™ 17 Microcentrifuge, Thermo Fisher Scientific, Waltham, MA, USA) and the precipitant was resuspended in water. As expected, the formation of particles was a result of the ionic interactions between the negative groups of TPP and the positively charged amino groups of CS. The same procedure and TPP ratios were also used for the preparation of particles with CS-tAcon, CS-Succ, CS-g-PHEA and CS-g-PAA derivatives. The ratio formulating the larger blank particles was chosen, hence, the selected ratios were namely: CS/TPP 4/1, CS-tAcon/TPP 7/1, CS-Succ/TPP 5/1, CS-g-PHEA/TPP 6/1 and CS-g-PAA 6/1.

For the preparation of drug-loaded particles, the same procedure was followed while the fluticasone propionate and salmeterol xinafoate ethanolic solution (fluticasone propionate/salmeterol xinafoate ratio was 2/1) was added in the CS or its derivatives solutions before the addition of the crosslinker. The final concentrations of drug solutions were 10, 20 and 30 wt% in total drug to CS polymeric matrix. The resulting solutions were magnetically stirred for 30 min followed by probe sonication (100 W, 30 kHz, Hielscher Ultrasonics, Teltow, Germany) for 2 min, to avoid aggregation. The particles were stirred for 4 h, and centrifuged at 11,000 rpm for 20 min. The non-entrapped drug was removed afterwards by washing the particles with ethanol and further centrifugation, while the precipitate was resuspended in deionized water and lyophilized by a freeze-drier system to obtain a dried product. 

### 3.4. Characterization of Materials and MPs

#### 3.4.1. Grafting Percentage and Grafting Efficacy

The final grafting percentage (GP, %) of the materials was calculated on the basis of the percentage mass increase in the final product relative to the initial mass of chitosan (Min and Mfin denote the mass of chitosan before and after the grafting process, respectively)

(1) $$\mathrm{GP}=\left(\frac{\mathrm{Mfin}-\mathrm{Min}}{\mathrm{Min}}\right)\times 100$$

Meanwhile, the grafting efficiency was calculated according to the equation

(2) $$\mathrm{GE}=\left(\frac{w2-w1}{w3}\right)$$

where w1, w2 and w3 are the initial weight of neat CS, the grafted weight of CS derivative and the weight of the added monomer respectively [67].

#### 3.4.2. Fourier-Transformed Infrared Spectroscopy (FTIR)

The chemical structures of modified CS and the synthesized MPs were determined with FTIR spectroscopy. FTIR spectra were recorded with an FTIR spectrometer (model FTIR-2000, Perkin Elmer, Waltham, MA, USA) using potassium bromide (KBr) discs (thickness of 500 μm after pressure). The spectra were collected in the range from 4000 to 400 cm^−1^ at a resolution of 4 cm^−1^ (total of 16 co-added scans), while the baseline was corrected and converted into absorbance mode.

#### 3.4.3. Nuclear Magnetic Resonance (NMR)

NMR spectra were recorded in a deuterated aqueous solution of acetic acid 2% *v*/*v*. An Agilent 500 spectrometer was utilized (Agilent Technologies, Santa Clara, CA, USA) at room temperature. Spectra were internally referenced with tetramethylsilane (TMS) and calibrated using the residual solvent peaks.

#### 3.4.4. Wide-Angle X-ray Scattering (XRD) 

X-ray powder diffraction (XRD) patterns were reported using an XRD-diffractometer (Rigaku-Miniflex II, Chalgrove, Oxford, UK), with a CuKα radiation for crystalline phase identification (λ = 0.15405 nm). All the samples were scanned over the range 2θ from 5° to 50° through a scan speed of 1°/min. 

#### 3.4.5. Dynamic Light Scattering (DLS)

The size of the resulting formulations was examined by dynamic light scattering (ZetaSizer 5000, Malvern company, Worcestershire, United Kingdom). One hundred microliters of the microparticle suspension were diluted in 900 μL of double distilled water. All measurements were executed in triplicate.

#### 3.4.6. Differential Scanning Calorimetry (DSC)

Thermal analysis studies were carried out by a Perkin–Elmer Pyris 1 differential scanning calorimeter (DSC) (Waltham, MA, USA) calibrated with indium and zinc standards in order to examine the crystallinity ratio of the active substances in the final formulations. About 5 mg of each sample were placed in sealed aluminum pans and heated up from 30 to 105 °C with a heating rate 20 °C/min in inert atmosphere (N_2_, flow rate 50 mL/min), were the current temperature was held for 1 min in order to remove the absorbed water, then samples were cooled to 30 °C and heated up again from 30 to 300 °C. The thermograms addressed were collected from the second heating scan.

#### 3.4.7. Thermogravimetric Analysis (TGA)

Thermogravimetric analysis (TGA) was performed in a Perkin–Elmer Pyris 1 TGA thermogravimetric analyzer (Waltham, MA, USA). Samples of 10 ± 0.5 mg were placed in alumina pans. An empty alumina pan was used as a reference. Heating was controlled by rotating temperature up to 600 °C in a 50 mL/min flow of N_2_. The heating rate was set at 20 °C/min and steady marks of sample temperature, sample weight, and heat flow were measured.

#### 3.4.8. Scanning Electron Microscopy (SEM)

Scanning electron microscopy (SEM) images were obtained with an electron microscope JEOL 2011 (Akishima, Tokyo, Japan). A small amount of each microparticles’ suspension was dropped to the holder and left to evaporate. The resulting samples were coated with carbon in order to administer a satisfying conductivity of the electron beam. Operating conditions were set at expediting voltage 20 kV, probe current 45 nA and counting time 60 s.

#### 3.4.9. Swelling Capacity

Regarding the controlled release systems, it was reported in previous studies that a material’s swelling ability affects the drug release mechanism [77]. Thus, swelling capacity for the four different CS derivatives was obtained. In detail, swelling ability was assessed by calculating the water sorption capacity in phosphate buffer with pH = 7.4 [77,78]. Swelling studies were carried out with sponges of neat and modified CS derivatives. Initially, each sponge was carefully weighed (W0) and immersed in buffer for several hours. At predetermined time intervals (i.e., 10, 15, 20, 30, 40, 60, 90, 120 and 180 min), the swollen samples were removed from the buffer, wiped with a filter paper to remove the excess surficial water, and weighed once more with the purpose of determining the swelling weight (W_n_). The percent weight alteration of the polymeric matrix throughout the swelling experiment (i.e., cumulative weight changes due to matrix swelling and erosion, S_(ti)_%) was measured through the following formula:

(3) $$S\left(\mathrm{ti}\right)\%\;=\left(\frac{\mathrm{Wn}-W0}{W0}\right)\times 100$$

#### 3.4.10. High-Pressure Liquid Chromatography (HPLC), Quantitative Analysis and Drug Loading

Quantitative analysis and drug loading were conducted utilizing a Shimadzu HPLC (Kyoto, Japan) system consisting of a degasser (DGU-20A5, Kyoto, Japan), a liquid chromatograph (LC-20 AD, Kyoto, Japan), an autosampler (SIL-20AC, Kyoto, Japan), a UV/Vis detector (SPD-20A, Kyoto, Japan) and a column oven (CTO-20AC, Kyoto, Japan). The samples were eluted with an isocratic method described by Jetzer et al., where simultaneous determination of both active compounds takes place. [79]. Specifically, the column was a type of CNW Technologies Athena C18, 120 A, 5 μm, 250 mm × 4.6 mm set at room temperature. In brief, the mobile phase consisted of ACN/H_2_O/TFA (58/42/0.1 *v*/*v*/*v*), while fluticasone and salmeterol xinafoate were detected at the wavelength of 225 nm. The flow rate through the HPLC system was 1 mL/min, whereas the adjusted injection volume was 20 μL. Quite sharp peaks were obtained at approximately 14 min (fluticasone propionate), 7.0 min (Salmeterol) and 4.5 min (xinafoate). Calibration curves were developed by diluting a 100 ppm stock methanol solution of each drug to concentrations of 0.01, 0.05, 0.1, 0.25, 0.5, 1.0, 2.5, 5.0 10.0, 20.0 and 30.0 ppm using ultrapure water. 

For the further demonstration of the drug loading capacity of the microparticles, 10 mg of the formulated particles was dissolved in 10 mL of aqueous acetic acid solution (1% *v*/*v*): methanol (50/50 *v*/*v*). The subsequent solution was stirred for 24 h and filtered through nylon filters (0.45 nm pore size). The microparticles’ yield, drug loading and drug entrapment efficiency (EE) were calculated using the following equations:

Yield (%) = [Weight of microparticles/Initial weight of polymer and drug] × 100(4)

Drug loading (%) = [Weight of drug in microparticles/Weight of microparticles] × 100(5)

EE (%) = [Weight of drug in microparticles/Initial weight of drug] × 100(6)

#### 3.4.11. In Vitro Dissolution Studies

In vitro release studies took place with the aid of DISTEK Dissolution Apparatus I (North Brunswick, NJ, USA) equipped with an autosampler (Evolution 4300, North Brunswick Township, NJ, USA) utilizing the basket method (USP I method). Microparticles placed into dialysis tubing cellulose membranes (MW cut-off 12,000–14,000, Servapor) were inserted in the apparatus baskets, while the dissolution operation was performed at 37 ± 1 °C, with a rotation speed of 100 rpm. The dissolution medium was 300 mL of a phosphate buffer adjusted at pH = 7.4, with the addition of Tween 80 (0.1% *w*/*w*). At predefined time intervals, 2 mL of aqueous solution was withdrawn from the release media and further analyzed for drug content by HPLC, as previously described.

## 4. Conclusions

In the present study, four different derivates of CS were synthesized after the modification of its backbone with trans-aconitic acid (t-Acon), succinic anhydride (Succ), 2-hydroxyethyl acrylate (2-HEA) and acrylic acid (AA). The success of the grafting was confirmed via FTIR and ^1^H-NMR spectroscopies. The new modified structures revealed a higher amorphous character, and higher swelling degrees than neat CS, due to the new functional groups that were incorporated in CS chains. Afterwards, CS and the formulated derivatives were utilized for FLU and SX microencapsulation, via ionotropic gelation. SEM images evidenced the spherical shape of the produced particles, while DLS measurements exhibited that all of them were ranging in the micrometric scale, which is appropriate for COPD formulations. FTIR spectra confirmed that some interactions take place between the characteristic groups of CS derivatives and the used drugs. The FLU and SX loading process in these formulations indicated increased drug encapsulation within the MPs’ networks with increasing drugs’ concentration. In a further step, XRD analysis showed that the inclusion of SX drug in all derivatives resulted in amorphous inclusions. FLU was also microencapsulated in amorphous form in the acrylic derivatives, while in CS, CS-tAcon and CS-Succ, the encapsulation was induced mainly in crystalline form. Finally, in vitro release studies revealed a substantial dissolution enhancement of both drugs from all CS derivatives. The release rate is much higher for SX drug due to its complete amorphization, following a similar release rate pattern in all derivatives. In the case of FLU, the release rate is higher only in CS-g-PHEA and CS-g-PAA derivatives, inducing them into advantageous candidates for SX and FLU co-encapsulation. From this study, it is clear that the four prepared CS derivatives can be used for microencapsulation of FLU and SX drugs, since all of them led to formulations characterized by enhanced drug release rates; nevertheless, CS-g-PHEA and CS-g-PAA derivatives possessed a more promising character due to their advanced FLU release profile.

## Figures and Tables

**Table 1:** Particle size distributions of various CS/Sodium Tripolyphosphate (TPP) w/w ratio for the blank particles determined by dynamic light scattering (DLS).

CS/TPP *w*/*w* Ratio	Particles Diameter (nm)				
	CS	CS-tAcon	CS-Succ	CS-g-PHEA	CS-g-PAA
2/1	292	904	505	272	566
3/1	364	237	276	276	640
4/1	450	423	392	297	550
5/1	427	389	518	381	584
6/1	359	597	503	833	972
7/1	304	957	491	560	829

**Table 2:** Size and zeta potential value of FLU/SX loaded microparticles of chitosan (CS) and CS derivatives.

Sample	Z-Average (d.nm)	Zeta Potential (mV)
CS-TPP-10% FLU/SX	1234	+51.9
CS-TPP-20% FLU/SX	1247	+47.8
CS-TPP-30% FLU/SX	1923	+45.7
CS-tAcon-TPP-10% FLU/SX	1210	+21.0
CS-tAcon-TPP-20% FLU/SX	1400	+21.4
CS-tAcon-TPP-30% FLU/SX	1943	+21.5
CS-Succ-TPP-10% FLU/SX	1451	+38.0
CS-Succ-TPP-20% FLU/SX	1507	+38.5
CS-Succ-TPP-30% FLU/SX	1708	+38.8
CS-g-PHEA-TPP-10% FLU/SX	754	+26.7
CS-g-PHEA-TPP-20% FLU/SX	1005	+22.6
CS-g-PHEA-TPP-30% FLU/SX	2216	+26.6
CS-g-PAA-TPP-10% FLU/SX	481	+34.3
CS-g-PAA-TPP-20% FLU/SX	438	+41.6
CS-g-PAA-TPP-30% FLU/SX	784	+39.4

**Table 3:** Salmeterol and fluticasone-loaded microparticles′ yield, drug loading and encapsulation efficiency.

Sample	Yield (%)	Drug Loading (%)		EE (%)
		Salmeterol Xinafoate	Fluticasone Propionate	
CS-TPP-10% FLU/SX	30.25	2.1	4.8	38.2
CS-TPP-20% FLU/SX	40.25	4.9	11.2	20.5
CS-TPP-30% FLU/SX	38.22	6.4	14.8	19.8
CS-tAcon-TPP-10% FLU/SX	34.23	1.68	12.01	55.47
CS-tAcon-TPP-20% FLU/SX	49.87	6.09	12.71	50.15
CS-tAcon-TPP-30% FLU/SX	50.21	7.56	11.79	47.8
CS-Succ-TPP-10% FLU/SX	29.12	4.77	13.44	17.1
CS-Succ-TPP-20% FLU/SX	35.12	6.29	19.8	11.25
CS-Succ-TPP-30% FLU/SX	42.54	5.08	25.2	11.13
CS-g-PHEA-TPP-10% FLU/SX	31.29	5.45	3.04	24.4
CS-g-PHEA-TPP-20% FLU/SX	39.75	8.6	10.28	19.5
CS-g-PHEA-TPP-30% FLU/SX	48.72	10.38	15.05	18.14
CS-g-PAA-TPP-10% FLU/SX	30.58	5.52	5.02	17.3
CS-g-PAA-TPP-20% FLU/SX	30.9	9.07	11.83	12.5
CS-g-PAA-TPP-30% FLU/SX	31.63	11.18	14.48	12.3

## Supplementary Materials

The following are available online, Figure S1: FTIR spectra of the four monomers used for the modification of CS’s backbone, Figure S2: SEM image of: (a) CS-TPP-10% FLU/SX, (b) CS-TPP-20% FLU/SX, (c) CS-TPP 30% FLU/SX, (d) CS-g-PHEA-TPP-10% FLU/SX, (e) CS-g-PHEA-TPP-20% FLU/SX, (**f**) CS-g-PHEA-TPP-30% FLU/SX, Figure S3: FTIR spectra of drug loaded CS nanoparticles, Figure S4: XRD diffractograms of salmeterol and fluticasone loaded nanoparticles, Figure S5: In vitro release rate (a) of salmeterol xinafoate from CS-FLU/SX microparticles, and (b) of fluticasone propionate from CS-FLU/SX microparticles.
