# Supplementary material for: Microencapsulation of Fluticasone Propionate and Salmeterol Xinafoate in Modified Chitosan Microparticles for Release Optimization
Source: Molecules. 2020 Aug 26;25(17):3888. doi: 10.3390/molecules25173888 (PMC7503413; doi:10.3390/molecules25173888)
Supplement: Supplementary file 1 [file molecules-25-03888-s001.pdf]

Article

Nanoencapsulation of Fluticasone Propionate and Salmeterol Xinafoate in modified chitosan nanoparticles for release optimization

Nina Maria Ainali, Eleftheria Xanthopoulou, Georgia Michailidou, Alexandra Zamboulis, Dimitrios N. Bikiaris*

Laboratory of Polymer Chemistry and Technology, Department of Chemistry, Aristotle University of Thessaloniki, Thessaloniki, Greece; ainali.nina@gmail.com (N.M.A.), elefthxanthopoulou@gmail.com (E.X.), michailidougeorgia18@gmail.com (G.M.), azampouli@chem.auth.gr (A.Z.)

* Correspondence: dbic@chem.auth.gr; Tel.: +30 2310 997812

Academic Editor: Mohamed Samir Mohyeldin

Received: 20 July 2020; Accepted: 24 August 2020; Published: date

Supplementary materials:

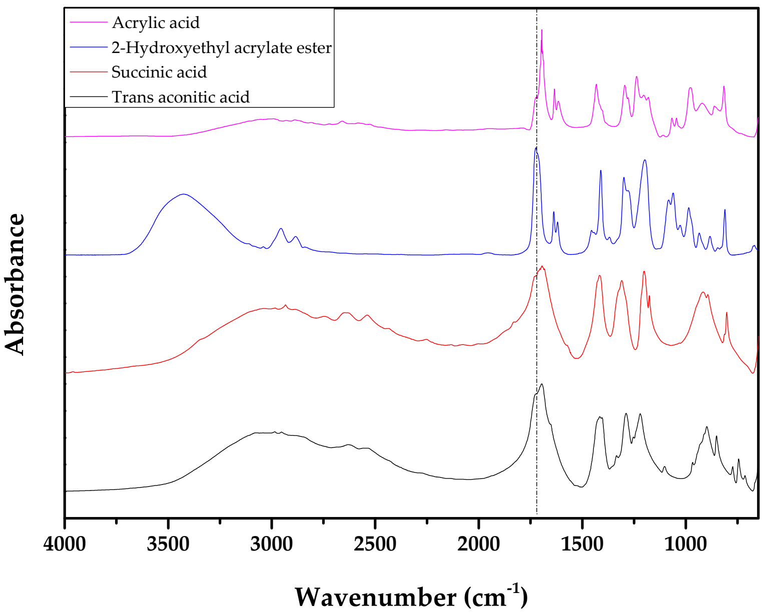

Figure S1. FTIR spectra of the four monomers used for the modification of CS's backbone.

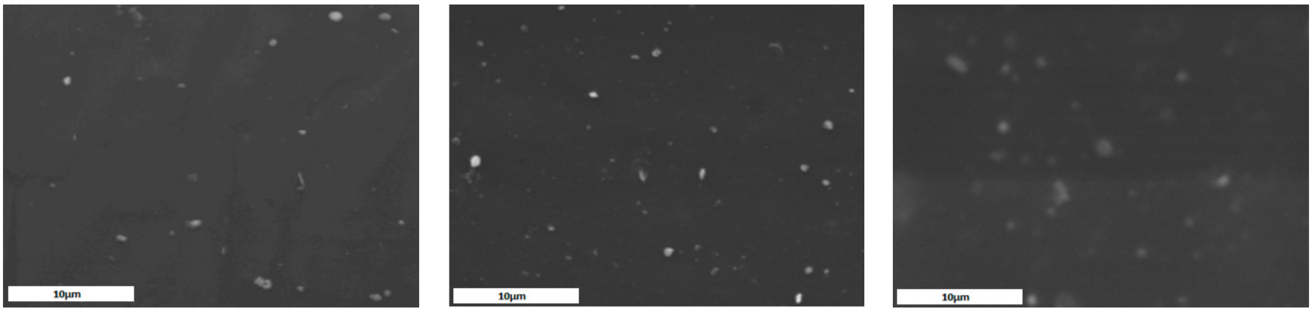

(a) (b) (c)

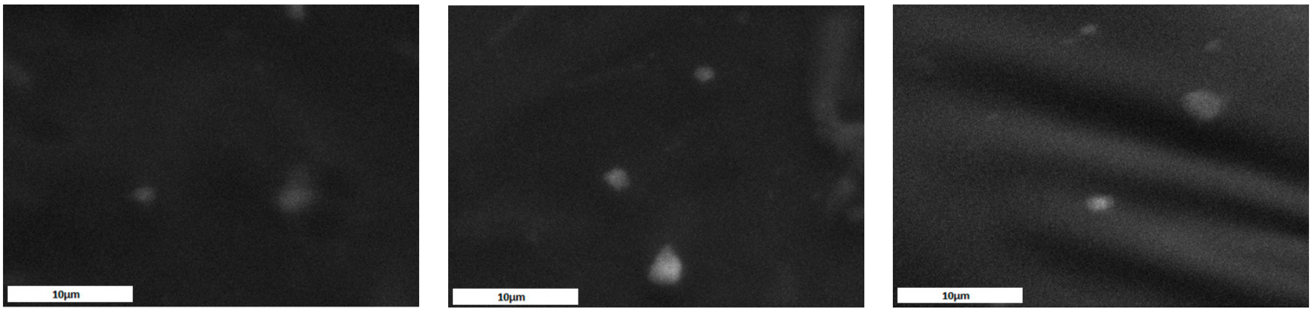

(**d**) (**e**) (**f**)

Figure 2. SEM image of: (**a**) CS-TPP-10% FLU/SX, (**b**) CS-TPP-20% FLU/SX, (**c**) CS-TPP 30% FLU/SX, (**d**) CS-g-PHEA-TPP-10% FLU/SX, (**e**) CS-g-PHEA-TPP-20% FLU/SX, (**f**) CS-g-PHEA-TPP-30% FLU/SX.

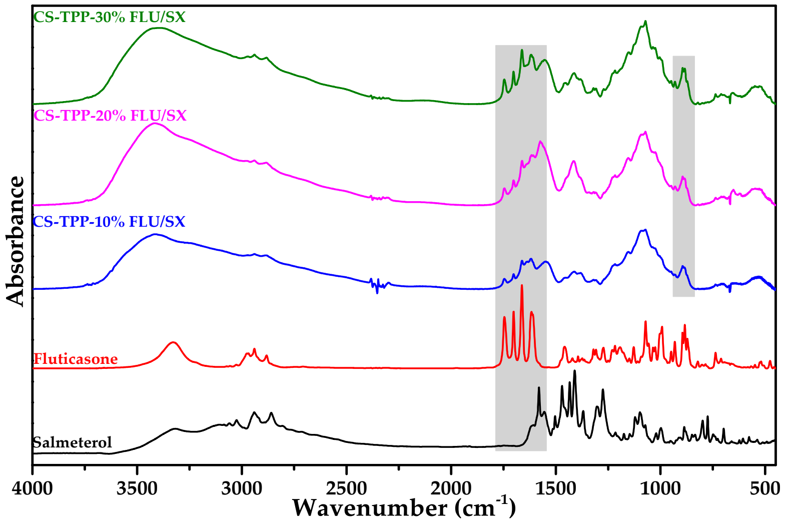

Figure 3. FTIR spectra of drug loaded CS nanoparticles.

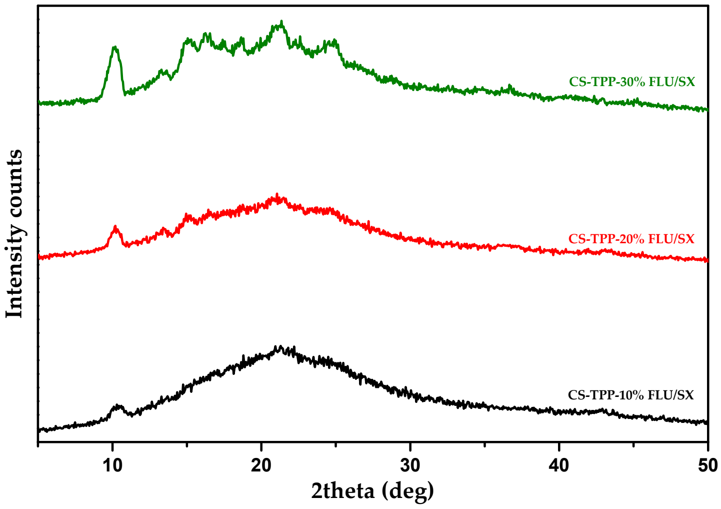

Figure 4. XRD diffractograms of salmeterol and fluticasone loaded nanoparticles.

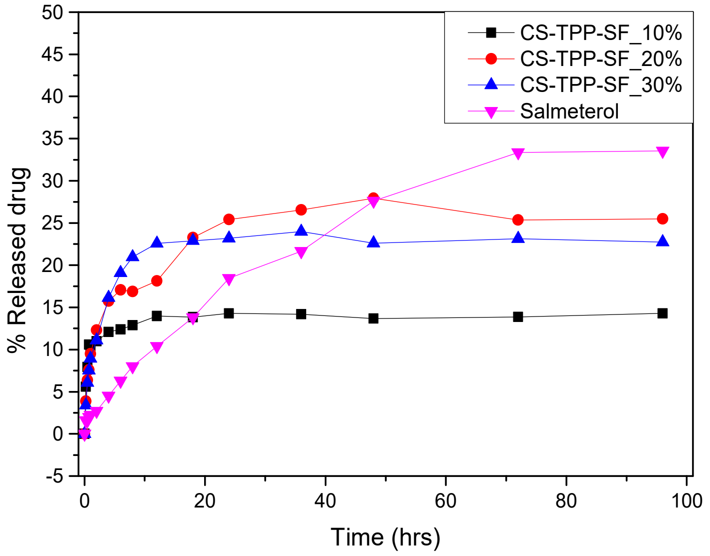

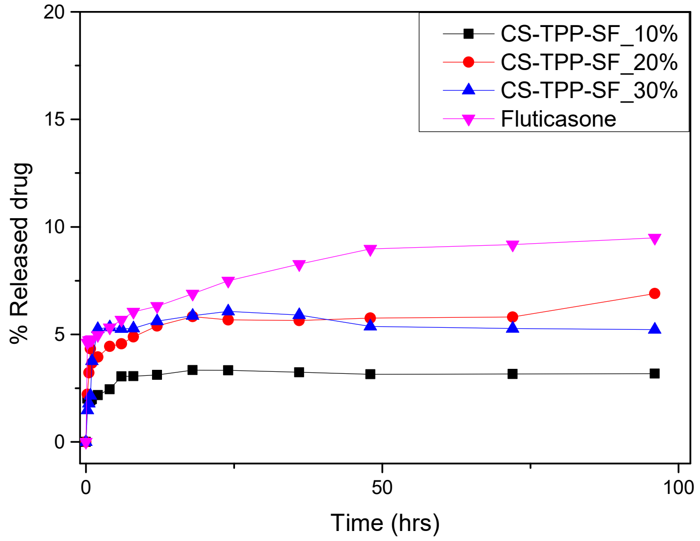

Figure 5. In vitro release rate (**a**) of salmeterol xinafoate from CS-FLU/SX microparticles, and **(b)** of fluticasone propionate from CS-FLU/SX microparticles.
